# Using community science to map western monarch butterflies (*Danaus plexippus*) in spring

**DOI:** 10.1002/ece3.10766

**Published:** 2023-12-27

**Authors:** Emily Erickson, Christopher Jason, Hannah Machiorlete, Lilianne de la Espriella, Elizabeth E. Crone, Cheryl B. Schultz

**Affiliations:** ^1^ Department of Evolution and Ecology University of California Davis Davis California USA; ^2^ School of Biological Sciences Washington State University Vancouver Washington USA

**Keywords:** annual lifecycle conservation, citizen science, community science, conservation of migratory animals, monarch butterfly, western monarch

## Abstract

Migratory animals follow seasonal cycles comprising linked phases often with different habitat requirements and demographic processes. Conservation of migratory species therefore must consider the full seasonal cycle to identify points limiting population viability. For western monarch butterflies, which have experienced significant declines, early spring is considered a critical period in the annual population cycle. However, records of western monarchs in early spring, when overall abundance is lowest, have historically been extremely limited. We used a community science initiative, the Western Monarch Mystery Challenge, to collect data on monarch distribution throughout the western United States between February 14th and April 22nd over 3 years. Using data from the Western Monarch Mystery Challenge and iNaturalist, we identified potential breeding habitat for western monarchs in early spring that spanned a large geographic area and several ecoregions. We observed monarchs in early spring that likely eclosed in the current year, suggesting that population expansion from overwintering sites reflects both movement and population growth. The number of records of western monarchs from early spring was higher during the Mystery Challenge (33.0/year) than earlier years (5.1/year). This study demonstrates the potential for and limitations of community science to increase our understanding of species at points in the life cycle when they are rare.

## Graphical abstract

Early spring is a critical period in the annual cycle of migratory monarch butterflies in the western United States. However, data on monarch distribution and demography during this time period has historically been extremely limited, presenting significant challenges to conservation management. Using a community science approach, this study presents quantitative data on western monarch distribution during the first part of the migration.

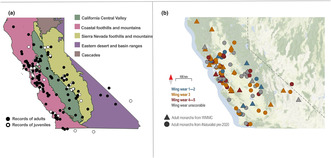

## 1 INTRODUCTION

Migratory animals go through seasonal cycles with distinct phases, often characterized by behavioral, morphological, and physiological shifts (Bhaumik & Kunte, 2020; Guo et al., 2011). These phases are inextricably linked to one another, such that processes in one phase (e.g., overwintering) impact those in a subsequent phase (e.g., breeding; Martin et al., 2007). Throughout their seasonal cycles, migratory animals can disperse broadly and may occupy diverse landscapes across vast geopolitical boundaries (Harrison et al., 2022). Effective conservation of migratory species therefore considers their ecology throughout their seasonal cycle and identifies spatiotemporal points limiting population viability (Flockhart et al., 2015; Martin et al., 2007; Schuster et al., 2019). In some cases, researchers have assumed that the places and times that host the largest population during a migratory cycle are the most important for population viability (Brown et al., 2017; Flockhart et al., 2015; Thogmartin, Wiederholt, et al., 2017). However, it may be that the point in the life cycle when a population is smallest is the most critical stage (Hewson et al., 2016; Ruegg et al., 2020).

The monarch butterfly (*Danaus plexippus*) is well known for its annual multi‐generational migrations (Flockhart et al., 2013). In North America, there are two geographically distinct populations of migratory monarch butterflies that are separated by the Rocky Mountains: a larger eastern population that overwinters in Mexico and a western population that overwinters along the Pacific coast (Freedman et al., 2021; Tuskes & Brower, 1978). Western monarchs gradually disperse inland from roughly late January through mid‐March (Tuskes & Brower, 1978). They then go through several breeding generations (Espeset et al., 2016). The generation produced in late fall then enters reproductive diapause before traveling back toward overwintering sites where they remain until the subsequent spring (Tuskes & Brower, 1978). Due to limited reproduction and stressors such as adverse weather events, there is an average decline in population size of about 40% from the start (roughly November) to the end (roughly March) of the overwintering period (Pelton et al., 2019; Tuskes & Brower, 1978). The first spring breeding generation therefore comprises only adults that have successfully survived both the fall migration and overwintering phases, meaning the population will be smallest in spring. It is still largely unknown where western monarch butterflies are during the first months after they leave overwintering sites, and it is also not known whether monarch breeding is common during this time period, which is before many western milkweeds (*Asclepias* spp., the monarch larval hostplant) emerge above ground (cf. Pelton et al., 2019).

Migratory western monarch butterflies have declined dramatically in recent years (Pelton et al., 2019; Schultz et al., 2017). In the 1980s, millions of monarch butterflies overwintered on the Pacific coast, primarily in California, with a small portion of the population in coastal Mexico. Since 2018, overwintering counts have consistently been <10% of estimates from the 1980s, with record low numbers—under 2000—in 2020 (Howard & Pelton, 2022; Xerces Society, 2022). In response, there has been significant investment in conserving and restoring monarch habitat in the West, including establishing stands of milkweed and planting native plants as nectar sources for adult butterflies (Western Association of Fish and Wildlife Agencies, 2019). Restoration efforts for eastern monarchs often focus on breeding habitat in summer and fall (Flockhart et al., 2015; Thogmartin, Wiederholt, et al., 2017). However, recent studies have found that population declines of western monarchs are greatest during the early part of the year, leading to the hypothesis that this is a point in the annual cycle that limits annual population growth (Espeset et al., 2016; Pelton et al., 2019). Early spring could be a limiting point in the seasonal cycle due to several factors, including habitat loss at overwintering sites (Espeset et al., 2016) or limitation of spring breeding habitat (Schultz et al., 2021). Western monarch populations are also at least as sensitive to climate conditions in spring as in late summer (Crone et al., 2019; Espeset et al., 2016). There are also practical implications to conserving spring breeding habitat. If the geographic range of the monarch butterfly population grows from spring to summer as the breeding season progresses, then restoring early season habitat will be more effective per unit area compared to later in the annual cycle (see Appendix S1).

Identifying early spring breeding habitat for monarchs poses significant challenges. Conventional monitoring during this time period would be extremely labor intensive due to the small population size at this time of year, likely spread over a large geographic range of potential spring breeding. Limited knowledge of monarch butterfly distribution and habitat use during early spring makes it difficult to identify regions for targeted monitoring. For example, before our study, sighting databases included fewer than 100 relevant observations during this period (see Section 2, below), compared to ~10,000 sightings used to evaluate eastern monarch distribution during fall (Momeni‐Dehaghi et al., 2021). This difference in data availability probably reflects in part the much larger population size of eastern monarch butterflies (nearly 100 million overwintering monarchs in the east compared to hundreds of thousands in the West; Schultz et al., 2017; Thogmartin, Wiederholt, et al., 2017). Sightings during summer and fall are also likely more common because monarch populations grow as the breeding season progresses (Flockhart et al., 2015), which further contributes to sparse data in spring.

In this study, we developed a community science program to collect data on early spring (mid‐February through April) monarch distribution across the western United States. Specifically, we (1) quantified the rate at which the western monarch butterfly population spread into the breeding grounds in spring and (2) compared the distribution and age structure (an indicator of potential breeding) of monarchs across ecoregions. We also assessed (3) whether the number and distribution of monarch records changed during the period after we initiated the Western Monarch Mystery Challenge (WMMC), a campaign to encourage community scientists to report monarch sightings in early spring. Finally, we used community science reports to evaluate (4) which milkweed species were reported as hosting monarch larvae during this early spring period.

## 2 METHODS

We compiled sightings of monarch butterflies during the early spring breeding period, which we defined as February 14 to April 22 (Valentine's Day to Earth Day—dates selected to increase participant engagement). Research grade observations with open locality data were pulled from iNaturalist (inaturalist.org) from 2008 to 2019, hereafter referred to as “historic data.” GBIF, Journey North, and eButterfly were also checked, but records on these sites were either incomplete based on our criteria (e.g., incomplete geocoordinates or no photographs), or duplicates of iNaturalist records. These passively collected iNaturalist observations were sparse (51 records during the early breeding season in the migratory breeding range; see Section 3). To encourage reporting of monarch butterfly observations during spring breeding, we established the WMMC in 2020. Participants were recruited to the WMMC via social media (Instagram, Facebook, Twitter) advertisements in English and Spanish and community outreach. Participants were asked to submit any photos that they had taken of a monarch butterfly during the specified date range to iNaturalist, the Western Monarch Milkweed Mapper, or directly to monarchmystery@wsu.edu. Communication with participants emphasized collecting observations of adult monarch butterflies to minimize the number of submissions of captively reared monarchs.

WMMC outreach focused on central and northern California and adjacent parts of Nevada, the area in which we most expected migratory monarch butterflies to breed in early spring in the West (Dingle et al., 2005). Nonmigratory populations of monarch butterflies are present in coastal urban gardens in California (Majewska et al., 2019). When we started this study, these nonmigratory populations were thought to be in the urban areas of southern California (e.g., Los Angeles and San Diego); therefore, we excluded all monarch butterfly observations south of Naples, CA (34.45, −119.92). During our study, it became obvious that nonmigratory monarch butterflies also occur in northern California cities, an apparently recent phenomenon (Crone & Schultz, 2021; James, 2021). These residents are associated with nonnative milkweeds that are evergreen in temperate climates (e.g., *Asclepias curassavica*) and encourage year‐round breeding behavior (Majewska et al., 2019). We did additional filtering of our data to remove possible resident individuals based on their occurrence in urban areas within the growing zone in which *A. curassavica* is evergreen, classified based on GIS land use data and USDA plant hardiness zones. Land use data (compiled in 2011) were obtained from the GAP/LANDFIRE National Terrestrial Ecosystems database (USGS, 2016) and USDA plant hardiness zones, derived from annual mean minimum temperature from 1976 to 2005, were obtained from the PRISM Climate Group (USDA, 2012). Both datasets were downloaded at a 30‐arc‐second resolution (approximately 1 km^2^) and the raster values for each monarch observation point in the challenge were extracted from the map using the “sample raster values” function in the “raster analysis” QGIS toolkit. Any points with a land use type of “Developed & Urban” and with an annual minimum temperature of 28.2°F were considered records of potential resident monarchs. Data on resident monarchs are limited; however, preliminary observations indicate only short‐distance local movement (E. Erickson, unpublished data). To ensure only migratory butterflies were in our dataset, any monarchs that were observed within 5 km of the closest possible resident monarch were also classified as possible residents and were removed from all analyses.

Because our goal was to understand spring migration, we removed any butterflies that were potentially overwintering. Overwintering monarchs often temporarily leave aggregations to forage on nearby nectar resources. For example, Sánchez‐Tlacuahuac et al. (2023) observed adults nectaring at experimental plots ~1.5 km from overwintering clusters. The exact movement distance of overwintering monarch butterflies is unknown, we therefore excluded monarch butterflies that were at or within 5 km of overwintering sites as a conservative measure that would exclude most overwintering butterflies.

Wing wear, which is a proxy for butterfly age (Malcolm et al., 1993), was scored for each image using a standard scale from 1 (newly eclosed, no wear) to 5 (heavily damaged, likely an older individual) (see Appendix S2 for wing wear classification information). Photographs in which the butterfly was too pixelated or far away to accurately classify were determined to be unscorable and were removed from the wing wear dataset but remained in the general distribution data. For records where the wing wear score was between two values, the lower score was assumed. Photographs from 2020 and 2022 and all historic records were scored by E. Erickson. Photographs from 2021 were scored by C. Jason. Consistency of wing wear scores evaluated by two separate individuals (E. Erickson and C. Jason) was assessed blindly on 166 out of 600 observations from 2021, including both migratory and urban adults. Of these, 47 we determined by one or both scorers to be unscorable. 74 out of the remaining 119 received identical scores from both observers (62%) and 42 differed by only one wing wear score (35%). Only three out of 119 scorable observation (2.5%) differed by more than one wing wear score between scorers. Observed butterflies were then broadly categorized based on when they likely eclosed as “current year” (wing wear 1–2) or “previous year” (wing wear 4 and 5). Butterflies with wing wear 3 were not able to be categorized as likely eclosed in previous or current year.

As a rough metric of the minimum distance traveled, we calculated the Euclidean distance of each observation from the nearest overwintering site. Locations of the Xerces Society's Western Monarch Thanksgiving counts (Xerces Society, 2022) were used to approximate overwintering site locations. Additional sites not included in the count (e.g., those on private land) and geocoordinate locations for all sites were obtained from the Xerces Society (E. Pelton, personal communication). Of these sites, all are along the Pacific Coast except for seven inland sites in Kern and Inyo counties, which are relatively small aggregations.

We tested whether monarchs expanded inland as the season progressed using a linear regression with the ln‐transformed distance to closest coastal overwintering site as the response variable and days since the start of the challenge as the predictor. We then tested whether breeding activity expanded inland as the season progressed using a binomial generalized linear model (GLM) with the response variable being whether or not an observed butterfly was wing wear class 1 or 2 and the predictor variables being the time since the start of the challenge each year, the distance from the closest overwintering site, and their interaction. It is impossible to determine where butterflies east of the inland sites in Kern and Inyo counties overwintered. Thus, we excluded observations east of longitude −117.92 for all analyses including distance from overwintering sites.

**FIGURE 1:**
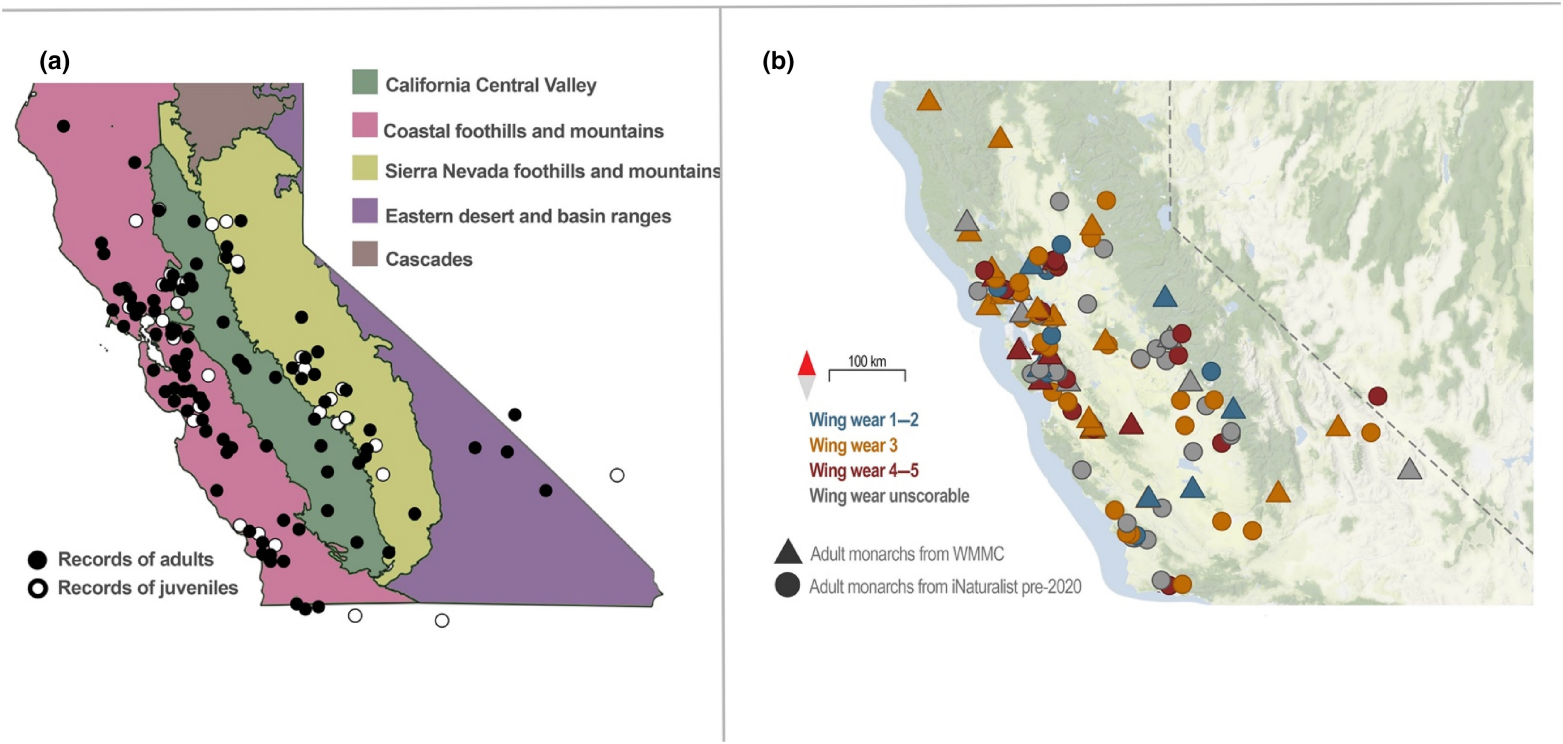
Monarch records within challenge region between February 14–April 22. (a) Distribution of records of all adult (black points) and juvenile (white points) monarchs across ecoregions from both historic data (2008–2019) and data from WMMC years (2020–2022) and (b) adult butterflies only, colored by wing wear score.

To evaluate the distribution of observations over the entire breeding period, we plotted all observations onto a map of custom ecoregions, adapted from EPA Level 3 ecoregions for California (US EPA, 2012). There were few records in Nevada, and all were assigned to the eastern desert and basin ranges ecoregion (see Figure 1 ecoregions). We assessed whether ecoregions differed in total number of reported monarch adults using negative binomial GLMs. To test which offset variables for sampling intensity best‐explained variation in the count data in each ecoregion, we first ran separate models with counts as the response and the ln‐transformed offsets of human population size, total land area total number of iNaturalist records for all species, and total iNaturalist records for Lepidoptera as the predictors (see below for details on how these data were collected). Using offsets in log‐link models leads to an analysis of counts per unit of the offset variable (cf. Zuur et al., 2009); therefore, these models evaluated whether the ratio of monarch butterfly counts to population size, to total land area, to iNaturalist counts of all species, or to iNaturalist counts of Lepidoptera was more consistent across years and ecoregions. Models were compared to each other and to the null model using AICc values. The predictor variable tested in the model with the lowest AICc (total land area; see Section 3) was then used as an offset subsequent modeling analyses.

To assess whether ecoregions varied in number of monarchs observed, we used a binomial GLM with summed counts of adult monarchs per year and ecoregion as the response variables, total land area as the offset, and ecoregion was the predictor. Similar to the analysis of distance vs. time, we tested whether breeding activity varied across ecoregions using a binomial GLM with the proportion of observations that were butterflies with wing wear 1 or 2 as the response and ecoregion as the predictor. To analyze whether the WMMC increased the number of records of adult monarchs in historically underreported regions, we used a negative binomial GLM with summed adults/year/ecoregion as the response variable, ecoregion and whether the year of collection was pre or during WMMC (2008–2019 vs. 2020–2022) as the predictor variables, and total land area of each ecoregion as the offset. To test whether total number of observations varied across time periods, we then ran an additional negative binomial GLM with the summed records of adults/year (across all ecoregions) as the response and whether the year of collection was pre or during WMMC as the predictor.

Observations of juvenile monarchs (eggs, caterpillars, pupae) submitted to iNaturalist were compiled and used as supplemental data to identify potential breeding habitat. We gathered all submissions within our study region, that is, California and Nevada north and west of (34.45, −119.92) from 2008 to 2022 in the months of February, March, and April. These data were processed in the same way as records of adults to remove potential residents and any geographic outliers. When possible, the hostplant was identified to species.

All analyses were conducted using R statistical software version 4.2.1 (R Core Team, 2023) and QGIS 3.22 (QGIS Development Team, 2023). Maps of observations were made using the ggmap package in R (Kahle & Wickham, 2013) and QGIS. Modeling analyses were conducted using the lme4 package (Bates et al., 2015) and the MASS package (Venables & Ripley, 2002) and models were evaluated using Type 2 “marginal” likelihood ratio tests, implemented using the Anova() function in the car package (Fox & Weisberg, 2019). Means were extracted from models using the emmeans package (Lenth, 2020) or by re‐running models with the intercept set at zero when an offset was included. Model fit was assessed using analysis of residuals and AICc values which were compared in the bbmle package (Bolker, 2020).

Total land area of each ecoregion was calculated in QGIS using the “add geometry attributes” function and total human population per ecoregion was calculated by multiplying the average population density for each ecoregion, calculated using the “zonal statistics” function on a human population density raster layer downloaded at 30‐arc‐second resolution (“Gridded Population of the World, Version 4 (GPWv4): Population Density, Revision 11”, 2018), by the total area. Total iNaturalist records of all species observed and of all Lepidoptera observed between the years of 2008–2022 submitted in the months of February–April with 1600 m coordinate accuracy were downloaded in August 2023 and October 2023 (respectively) from The Global Biodiversity Information Facility (“GBIF Occurrence Download”, 2023) and points were plotted onto ecoregions in QGIS. Total iNaturalist records (all species and all Lepidoptera) for all years in each ecoregion were calculated using the “count points in polygon” function in QGIS.

## 3 RESULTS

Historic iNaturalist data contained 83 records within challenge dates and geographic range outside of overwintering sites and with open locality information. Of these 83 records, 51 were likely adult migratory butterflies—that is, outside of urban areas with year‐round tropical milkweed. The earliest records were in 2008 (when iNaturalist launched). 40 of these records were scorable for wing wear; 6 of the 40 were migratory butterflies with wing wear class 1 or 2, eight were migratory butterflies with wing wear class 4 or 5, and 26 were of butterflies with wing wear class 3 (Table 1).

**TABLE 1:** Number of monarch records on iNaturalist prior to the start of the Western Monarch Mystery Challenge and number of records of monarchs gathered during the Mystery Challenge, by ecoregion and wing wear class.

	Coastal foothills and mountains	California Central Valley	Sierra Nevada foothills and mountains	Eastern desert and basin ranges
# adults pre‐WMMC (2008–2019)				
Wing wear 1–2	3	1	2	0
Wing wear 4–5	7	1	0	0
Wing wear 3	20	1	4	1
Unk. wing wear	7	0	3	1
# adults during WMMC (2020–2022)				
Wing wear 1–2	5	2	2	0
Wing wear 4–5	10	5	3	1
Wing wear 3	18	7	4	1
Unk. wing wear	17	4	20	0
Human population size (2020)	9.2e+6	7.2e+6	1.1e+6	4.3e+5
Total area of each ecoregion (km2)	1.03e+5	4.6e+4	7.1e+4	7.6e+4
iNaturalist records for Feb‐Apr (2008–2022)—all species	4.8e+5	5.4e+4	3.7e+4	1.8e+4
iNaturalist records for Feb‐Apr (2008–2022)—all Lepidoptera	19,413	1553	1683	489

*Note*: Values of offset variables for each ecoregion are listed on the bottom three rows.

Across 3 years of the WMMC, we collected 1736 total records of monarchs. 368 of these records were submitted directly via email, 1113 were gathered via iNaturalist, 251 from the Western Monarch Milkweed Mapper, and four from other social media platforms (eg. Instagram). Of these, 1645 of which contained locality data and 992 were within the dates and geographic range of the WMMC—that is, California and Nevada north and west of (34.45, −119.92). 364 were at least 5 km from overwintering sites. We removed 265 potential resident butterflies (those that were in urban areas where tropical milkweed is evergreen). Ultimately, only 99 were determined to be likely migratory individuals within the challenge region: 11 from 2020, 10 from 2021, and 78 from 2022. Of the 99 records of migratory butterflies, 66 were scorable for wing wear. 12 of the 66 records of migratory butterflies were wing wear class 1 or 2, 19 records were of migratory butterflies were wing wear class 4 or 5, and 35 records were of wing wear class 3 (Table 1).

**FIGURE 2:**
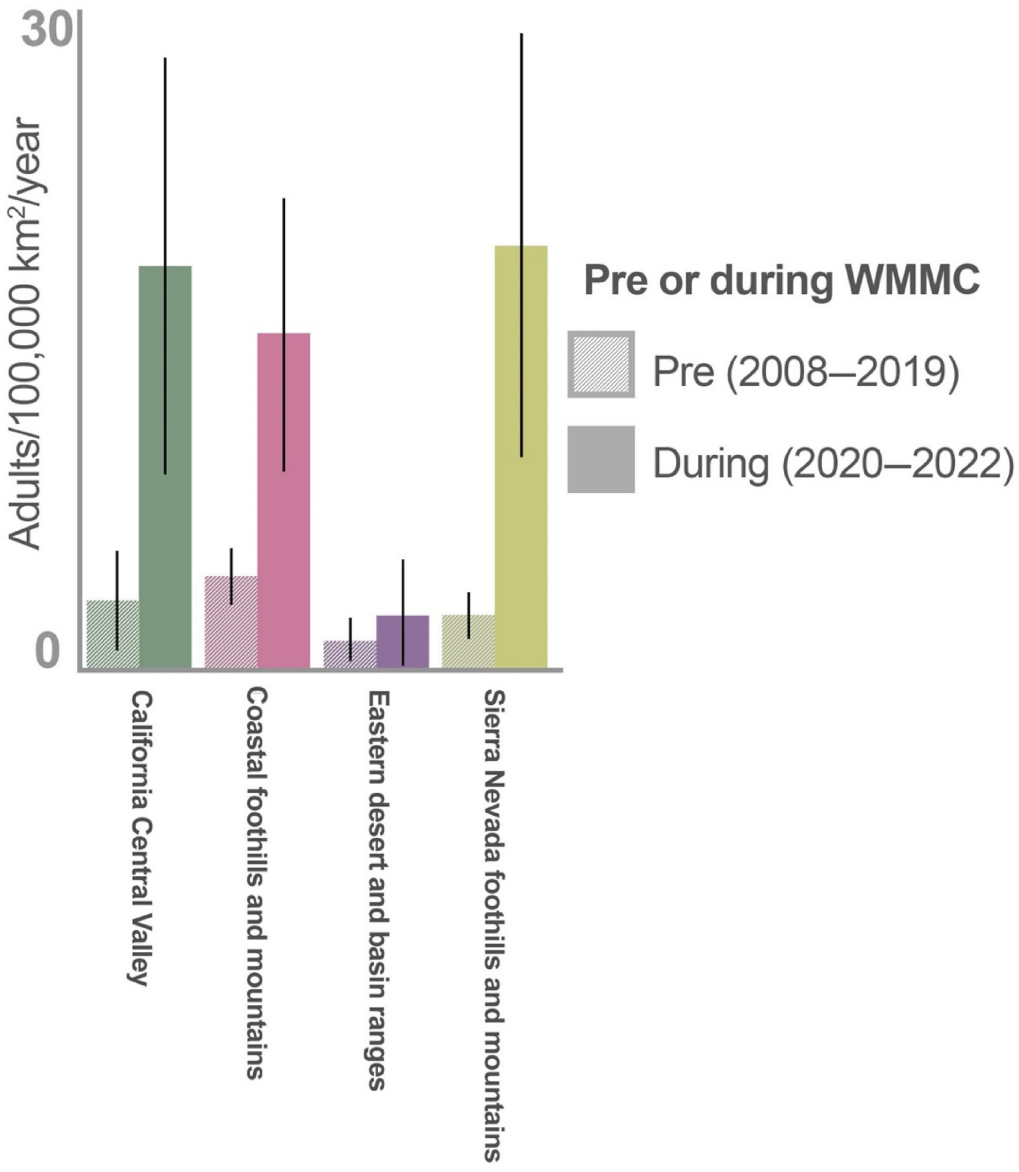
Average number of monarch adults per 100,000 km^2^ recorded per ecoregion before and during WMMC. Mean adults/100,000 km^2^ ± SE in years before and after the WMMC. Total number of monarch records during the early spring period increased significantly in years following WMMC; however, there was no significant interaction between ecoregion and time period.

Total land area was the best offset for explaining variation in count data (AICc = 147.6), compared to human population (AICc = 156.7), iNaturalist counts for all species (AICc = 158.8), iNaturalist counts for Lepidoptera (AICc = 159.2), and a null model with no effort covariates (AICc = 148.0). After accounting for differences in land area, adult butterfly counts did not differ significantly among ecoregions (*χ* ^2^ = 4.01, *p* = .26, DF = 3, *n* = 25). Although not statistically significant, the number of monarch sightings per area was noticeably lower in the eastern desert and basin ranges (1.76 adults per 100,000 km^2^, 95% CI = [0.40, 8.41]) than the Sierra Nevada foothills and mountains (7.63 adults per 100,000 km^2^, 95% CI = [3.71, 18.00]), the coastal foothills and mountains (7.67 adults per 100,000 km^2^, 95% CI = [4.37, 14.80]), or the Central Valley (11.29 adults per 100,000 km^2^, 95% CI = [4.41, 36.71]). The number of records per year across ecoregions was significantly higher after the start of the WMMC (*χ* ^2^ = 14.50, *p* < .01, df = 1, *n* = 13). There weas no significant interaction between ecoregion and time period (before or after the WMMC) (*χ* ^2^ = 1.44, *p* = .70, df = 3, *n* = 25) (Figure 2).

**FIGURE 3:**
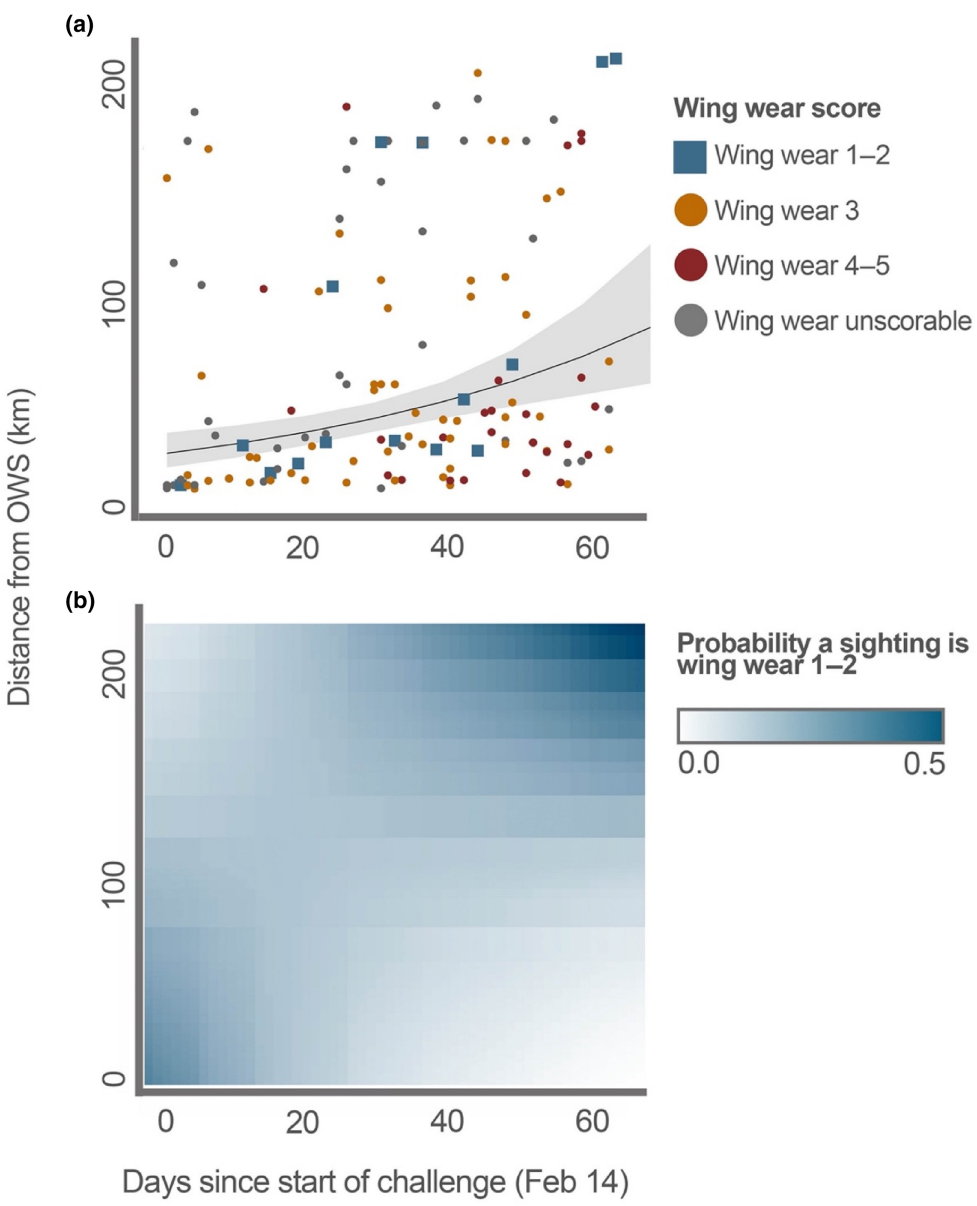
Predicted distance from overwintering site as a function of time. (a) Prediction plot from a linear model (LM) of distance from the closest overwintering site (OWS) as a function of time since the start of the WMMC (which runs from February 14–April 22 annually). Monarch records from this study are overlaid as points colored by wing wear. Shading represents 95% CI. (b) predicted interaction between distance from overwintering site and time since the start of the WMMC on the proportion of wing wear 1 and 2 butterflies. Breeding starts early in the season near the overwintering sites and progresses inland by later in the spring.

The distance of adult migratory butterflies from the overwintering sites increased through time (days since start of WMMC in each year) (*F* = 14.27, *p* < .01, df = 1, *n* = 146) (Figure 3a). There was a close to significant interaction between distance to overwintering site and time on the proportion of monarchs with wing wear 1–2 (monarchs that likely eclosed in the current spring; *χ* ^2^ = 2.96, *p* = .09, df = 1, *n* = 95); the proportion of wing wear 1–2 butterflies further from overwintering sites was higher later in the season (Figure 3b). Ecoregion was not a significant predictor of the proportion of observations that were wing wear class 1 or 2 (i.e., the proportion of butterflies likely to have eclosed during the current spring; *χ* ^2^ = 2.69, *p* = .44, df = 3, *n* = 98).

**FIGURE 4:**
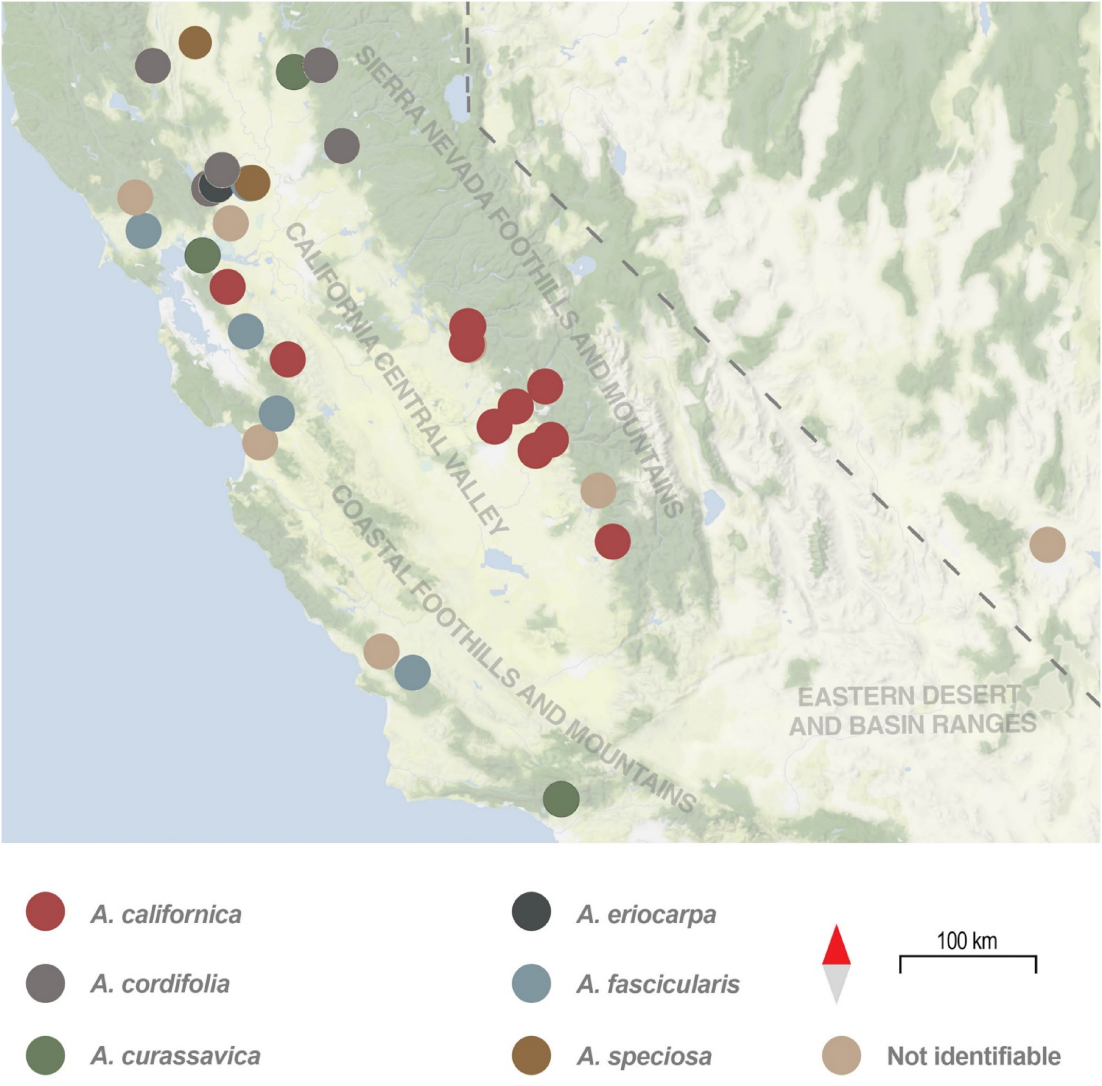
Distribution of larval and egg records from iNaturalist. Records are color coded by milkweed hostplant. Monarchs may use several different species of milkweeds during the first part of their breeding season including early‐emerging native species such as *Asclepias cordifolia*, *A. californica* and *A. eriocarpa*.

iNaturalist data included 45 records of juvenile monarchs that had been submitted in WMMC months and were outside of urban spaces with year‐round tropical milkweed (Figure 4). Of these, there was only one record each of eggs and pupae, and 43 records of larvae. The Sierra Nevada foothills and mountains and the coastal foothills and mountains had 17 and 19 records, respectively, and the Central Valley had six. Three records were in the eastern desert and basin ranges. Hostplant species could be identified in 35 of the 44 larval and egg records, with 15 records on *A. californica*, 6 on *A. cordifolia* and *A. fascicularis*, 4 on *A. curassavica*, and 2 records each on *A. eriocarpa*, and *A. speciosa* (see Figure 4).

## 4 DISCUSSION

Prior to the start of the WMMC, data on western monarch distribution during the early spring were extremely limited. From 2020 to 2022, we observed a ~2‐fold increase in reports of monarchs in California and Nevada during this critical period in the annual cycle. This increase may be due, in part, to increased usership of online community science databases such as iNaturalist. For comparison, there was a 1.6‐fold increase in all (monarch and non‐monarch) iNaturalist records, both globally as well as California only in 2008–2019 versus 2020–2022 (inaturalist.org). Certainly, we are unable to distinguish whether this increase resulted directly from our campaign or from an increase in attention to monarch butterflies overall. Nonetheless, it is consistent with the notion that the WMMC increased our knowledge of monarch butterflies during early spring breeding, when they are most sparsely distributed and therefore most difficult to observe.

Overall, the number of records gathered during the WMMC is consistent with annual monarch population counts at overwintering sites during those years (2020–2022). That is, only 29,436 and 1899 butterflies were recorded in November 2019 and 2020 Thanksgiving counts, respectively, and 247,246 recorded in 2021 Thanksgiving counts (Xerces Society, 2022). Correspondingly, our data showed similarly low numbers in 2020 and 2021 (the springs following the 2019 and 2020 Thanksgiving counts) and a nearly 8‐fold increase in recorded migratory monarchs in 2022. The increase in sightings reported in 2022 may reflect many factors other than monarch population size, such as increased public awareness of monarchs following the crash. Still, our results support the hypothesis that the western monarch population grew during the summer breeding season in 2021 rather than alternative hypotheses such as an influx of individuals in early spring from urban gardens (James, 2021) or from unknown inland overwintering sites (Taylor, 2022).

Our study also supports the notion that monarch butterfly population expansion in early spring is a gradual process reflecting both population growth and inland movement. Tuskes and Brower (1978) showed that monarchs begin gradually dispersing from overwintering sites in January, with mated females likely being the first to leave. Dispersal then continues at increasing rates until roughly March, when most of the colony has left overwintering sites. However, it was largely unknown whether western monarchs were breeding or simply moving to summer breeding sites following dispersal from overwintering sites. Our data suggest that western monarch breeding begins early in the year close to overwintering sites and expands inland and covers a wide geographic range as far east as the Sierra Nevada Mountains. Nonetheless, data are still limited during this time period. We did not obtain enough observations to distinguish the use of different ecoregions, let alone finer‐scaled habitat characteristics. Furthermore, we caution that even the statistically supported relationships in our models may not be quantitative predictors of monarch breeding locations through time. Linear modeling is a powerful tool for drawing qualitative inference from sparse data (i.e., directionality of relationships, in this case greater distance of all monarchs and the proportion of young monarchs through time). However, monarch movement through the landscape is not necessarily linear, limiting quantitative interpretation. Further monitoring, and, ideally, systematic sampling, would be needed to resolve the functional shape and limits of the data.

Another caveat to our conclusions is that we inferred breeding primarily from the presence of young monarch butterflies at breeding sites. Wing wear is not a perfect proxy for age, and it is possible in theory that some butterflies survived the winter with low wing wear. This possibility is unlikely in practice since monarch butterflies at overwintering sites typically have high wing wear (Malcolm et al., 1993; Miller et al., 2012). It is also possible that young butterflies moved from their natal sites before they were observed, although movement also tends to be associated with an increase in wing wear (Korkmaz et al., 2022; Miller et al., 2012). In our wing wear classification (see Appendix S2), adults with wing wear 1 would have eclosed within several hours (Schroeder et al., 2020). Our data included one observation of a wing wear class 1 butterfly, which was located in Sierra Nevada foothills, suggesting that at least some butterflies were born inland and far from coastal overwintering sites during this early spring period.

Native western milkweeds species vary in their phenologies and ecologies, meaning the monarch breeding landscape changes through space and time across the migratory cycle. In our data, monarch larvae were observed primarily on early season milkweeds: *Asclepias cordifolia*, *A. eriocarpa*, and *A. californica* (Figure 4). The latter two milkweed species are largely associated with dry grasslands or woodlands and the former with rocky slopes in regions with moderate precipitation (CalScape.org). These early season milkweeds are often found in foothill habitats (Dilts et al., 2019) and are some of the first to break dormancy in the spring. These early season milkweeds are also less abundant overall compared to milkweeds that mature in summer and fall (e.g., *A. speciosa*, *A. fascicularis*) (Dilts et al., 2019) and are dormant or declining late in the season when monarch populations are largest. Previous studies have characterized the geographic origins of monarchs at overwintering sites (Yang et al., 2016), often with the implication that the fall breeding generations and their associated milkweed hosts are the most important for final population size (Flockhart et al., 2017; Thogmartin, López‐Hoffman, et al., 2017). However, our results highlight the fact that restoration efforts will also need to include early‐emerging milkweed species in order for populations to grow throughout the summer (see also population growth models in Appendix S1). Our results also suggest that such habitat is likely to be used over the wide geographic area including the Coast Range, Central Valley, and Sierra Foothills regions.

One challenge of presence‐only community science data is accounting for sampling effort that may bias interpretation of results (Geldmann et al., 2016; Isaac et al., 2014). Of the four variables we tested in this study to account for sampling intensity within ecoregions (total land area, human population, total iNaturalist records, and iNaturalist records of Lepidoptera), the area of the ecoregion best‐explained variation in monarch counts. This may reflect unequal engagement and participation by people in community science initiatives across ecoregions with varying population sizes, perhaps due to differences in access to relevant information and resources to participate (e.g., Sicacha‐Parada et al., 2021). Another interpretation of these results is that iNaturalist activity is not a reasonable proxy for participation in the WMMC, which pulls data from several sources including iNaturalist. It may therefore be that through outreach efforts, we successfully engaged individuals in the WMMC who would not otherwise contribute to community science databases. Alternatively, it could be that trends in recording of charismatic species such as monarch butterflies on public databases or collections are not equivalent to those of less publicly visible species or overall biodiversity (Di Cecco et al., 2021; Wepprich, 2019).

In conclusion, this study provides the first quantitative information about the geographic distribution of western monarch butterflies in early spring, likely a critical point in the annual life cycle. Our study also supports the role of outreach to community scientists in helping observe charismatic species when they are sparse. Still, the number of observations in this study is lower than one might expect for a widespread and charismatic species (cf. Momeni‐Dehaghi et al., 2021; Ries et al., 2015). While community science is an effective tool for engaging the public and increasing sampling effort, it is not a panacea for limited data (Devictor et al., 2010; Kremen et al., 2011). If we want to understand the differences in habitat use across these regions, we may need to employ systematic, effort‐controlled, surveys. Alternatively, we may be able to infer habitat quality from detailed observations of movement, reproduction, and larval survival of individual butterflies during this time period, rather than relying on inference from broad spatial distribution data. As community science becomes more widely used in ecology, it is important to recognize its limitations as well as its strengths. Nonetheless, these observations provide a strong foundation for future research, and are the best available data to date for focusing management efforts for monarchs in the West during this period.

## AUTHOR CONTRIBUTIONS

**Emily Erickson:** Data curation (equal); formal analysis (equal); project administration (equal); writing – original draft (lead); writing – review and editing (lead). **Christopher Jason:** Data curation (equal); project administration (equal). **Hannah Machiorlete:** Data curation (equal); project administration (equal); writing – review and editing (equal). **Lilianne de la Espriella:** Project administration (equal); writing – review and editing (equal). **Elizabeth E. Crone:** Conceptualization (equal); formal analysis (lead); funding acquisition (equal); methodology (equal); writing – original draft (supporting); writing – review and editing (equal). **Cheryl B. Schultz:** Conceptualization (lead); funding acquisition (lead); investigation (equal); methodology (equal); project administration (equal); resources (equal); writing – original draft (supporting); writing – review and editing (equal).

## FUNDING INFORMATION

This project was supported by Monarch Joint Venture, the Xerces Society for Invertebrate Conservation, and the Western Association of Fish and Wildlife Agencies (WAFWA), NSF Rapid Response Research (RAPID), Google, and Conservation, Research and Education Opportunities International (CREOi).

## CONFLICT OF INTEREST STATEMENT

The authors declare no competition of interest.

### OPEN RESEARCH BADGES

This article has earned an Open Materials badge for making publicly available the components of the research methodology needed to reproduce the reported procedure and analysis. All materials are available at https://doi.org/10.5061/dryad.rjdfn2zj3; https://datadryad.org/stash/share/‐Ikmes9xtpSR1j6PSdQUeySJvD3iSrrkxvfdWUIEKSM.

## Supporting information


Appendix S1.

## Data Availability

The data is stored in Figshare at the following link (https://doi.org/10.6084/m9.figshare.24596202).
