# Supplementary material for: Using community science to map western monarch butterflies (Danaus plexippus) in spring
Source: Ecol Evol. 2023 Dec 27;13(12):e10766. doi: 10.1002/ece3.10766 (PMC10752247; doi:10.1002/ece3.10766)
Supplement: Supplementary file 1 — Appendix S1. [file ECE3-13-e10766-s001.docx]

**Appendix**

**1. *Importance of early spring breeding for monarch butterfly population growth***

To illustrate the importance of early spring breeding, we use a simple population model of the full annual life cycle (Figure S1). We assume that the population grows exponentially through four successive generations (cf. Crone & Schultz 2022, discussed further below), leading to the following equation for the annual population growth rate, Λ:

**eq. (S1)**

| $\Lambda= \lambda_{1}\times\lambda_{2}\times\lambda_{3}\times\lambda_{4}\times\psi_{fall}\times\psi_{winter}$ |  |
| --- | --- |

where $\lambda_{i}$ is the growth rate from generation i-1 to i, and $\psi_{x}$ represents survival during non-breeding parts of the life cycle. Because this is a simple multiplicative model, all of the parameters have the same elasticity (proportional sensitivity, see, e.g., Caswell, 2001; Morris & Doak 2002). This fact reflects the commutative property of multiplication (i.e., A×B×C = C×A×B). In other words, a 10% change in growth rate during the first generation has the same effect on annual population growth rate as a 10% change in the growth rate during the final generation.

**Figure S1**. Full annual life cycle typical of migratory monarch butterflies

Western monarch butterflies have an expanding range during summer breeding (Schultz et al. 2019, Figure S2), which means that each generation uses a larger geographic area. If the population growth rate of each generation were proportional to habitat quality in each generation, then a 10% improvement of the small area used for early spring breeding would have the same impact on annual population growth rate as a 10% improvement in the large geographic area used for late summer breeding. Similarly, an

addition of (say) 10 Ha of breeding habitat in the early spring breeding range would have a much larger impact than a 10 Ha addition of breeding habitat in the late summer breeding range because it would affect a much larger proportion of the population during that part of the annual life cycle. Therefore, early spring habitat restoration has a much larger impact per habitat area on population growth.

Of course, there are many caveats to these simple calculations. If monarch butterflies were strictly limited by carrying capacity during the last breeding generation (i.e., even a single monarch butterfly could produce enough offspring to find and use all available host plants), then only the habitat availability for the last breeding generation would affect the annual population growth rate. However, it seems likely that monarch butterflies are limited by host plants through a density-independent mechanism (search time limitation) in their western migratory range (see Crone and Schultz 2022). Empirical tests of habitat limitation at different stages of the migratory monarch butterfly’s annual life cycle would be an interesting but challenging area of future research. In the meantime, this model adds one more line of evidence to the potential importance of restoring monarch habitat during the time of year when populations are smallest.

**Figure S2**. Seasonal variation in area used by Western monarch butterflies for breeding (reproduced from Schultz et al. 2019)


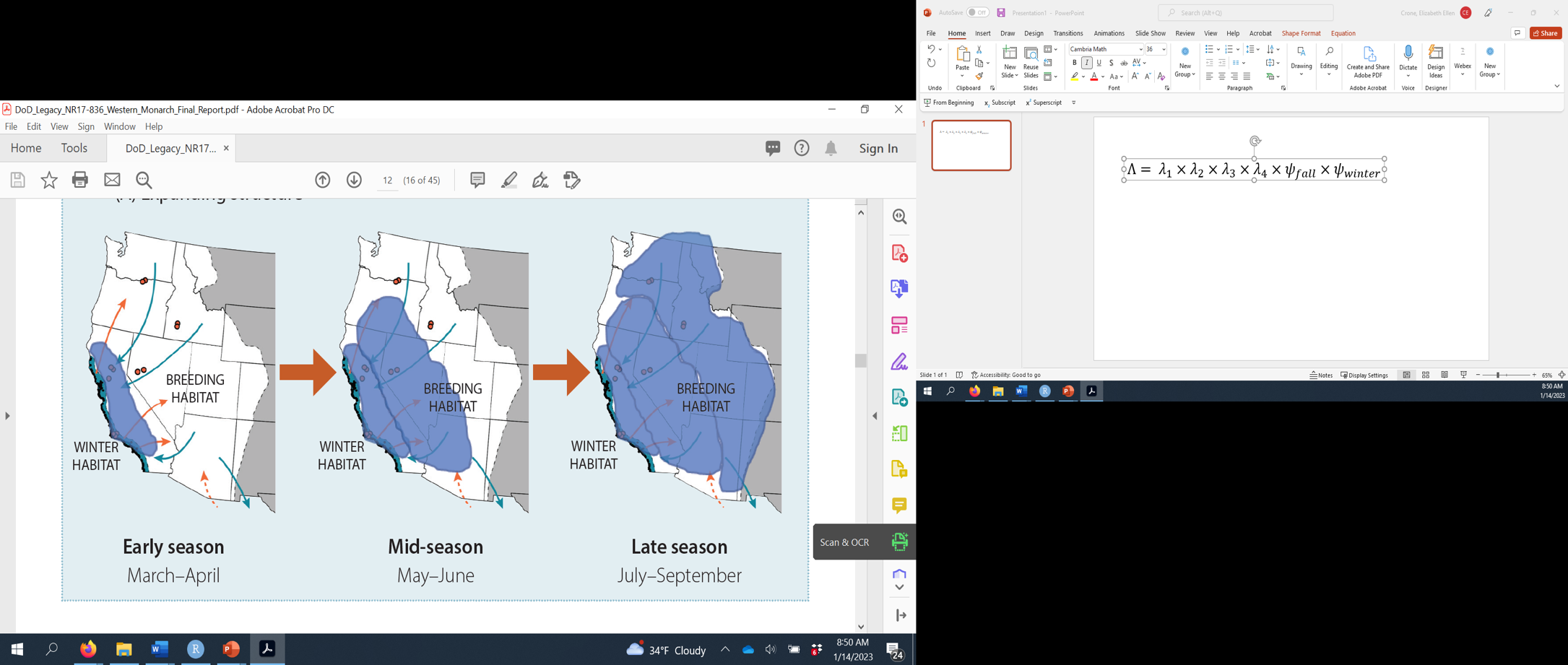


**2. *Wing wear scoring categories***

**Condition 1:** An adult monarch is scored as wing wear 1 if it appears very recently eclosed - i.e. the wings have no visible tears or scale loss (with the exception of cases where scales are lost from not eclosing correctly). Folds are after visible near margin of the wings.

**Condition 2:** An adult monarch is scored as wing wear 2 if it has no visible damage to wings such as tears or scale loss, but the folds near the margin of the wings are no longer visible.

**Condition** **3:** An adult monarch is scored as wing wear 3 if it has minor damages or tears on the wings but these do not extend past the black border on the outer margin of the wings. These butterflies will often have light to moderate scale loss and the black markings on the wing will appear lighter in color compared to the black of the head and thorax.

**Condition 4:** An adult monarch is scored as wing wear 4 if there are moderate wing damages such as tears that pass through the black border on the margin of the wings. These butterflies will have moderate to high scale loss and black markings will be faded to grey and are much lighter than the black of the thorax and head.

**Condition 5:** An adult monarch is scored as wing wear 5 if it has major wing damage and at least 1/3 of the total wing area is lost. Wings will appear very faded to almost translucent due to a high degree of scale loss.

| Table S1 ***Sample photos from data demonstrating wing wear conditions*** | | |
| --- | --- | --- |
| **Wing wear 1**  *photo by iNaturalist user sagecat* | **Wing wear 2**  *photo by iNaturalist user aliecat* | **Wing wear 3**  *photo by iNaturalist user robinellison* |
| 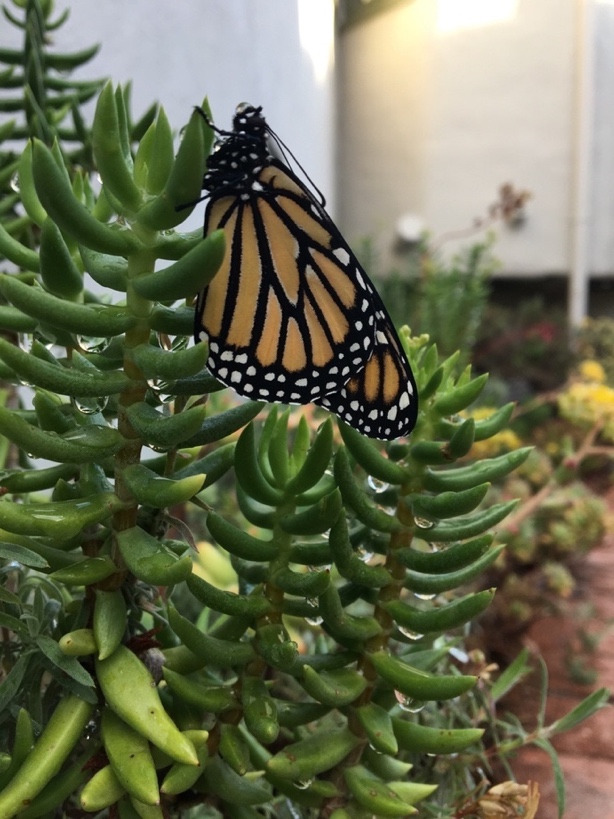 | 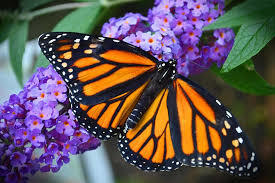 | 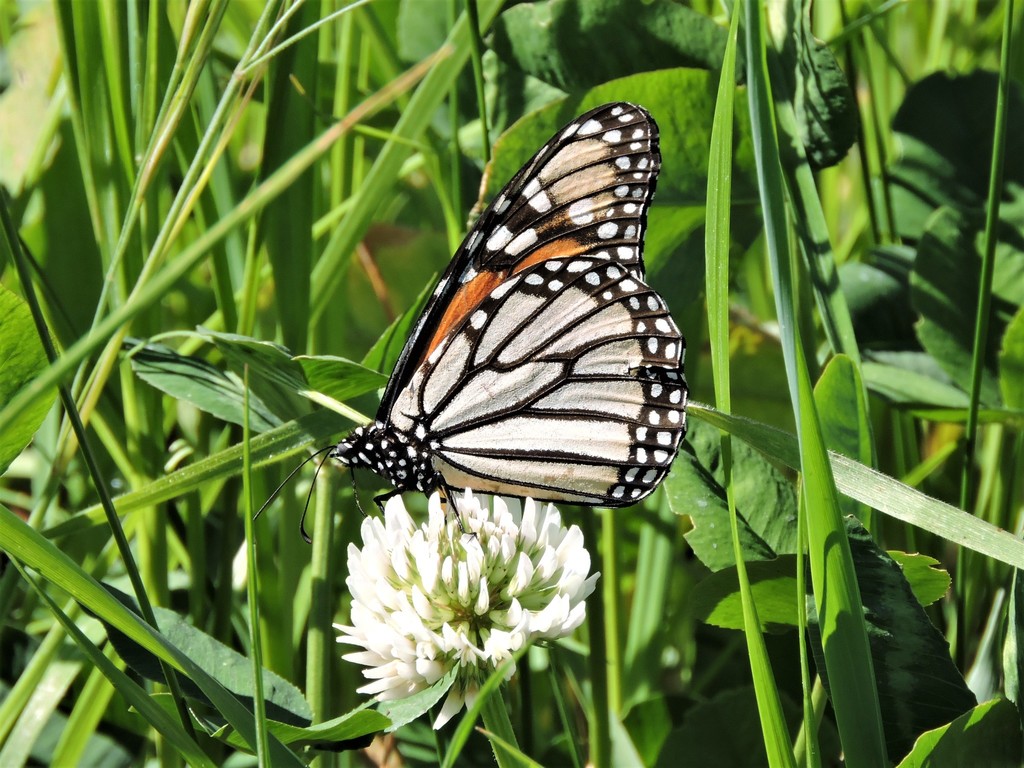 |
| **Wing wear 4**  *photo by iNaturalist user djrawlson* | **Condition 5**  *photo by iNaturalist user icosahedron* |  |
| 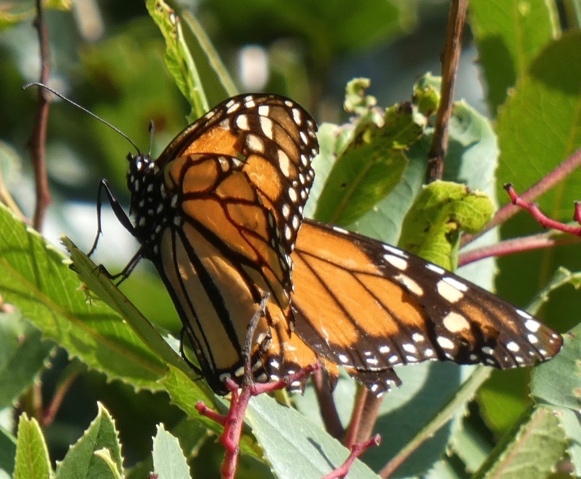 | 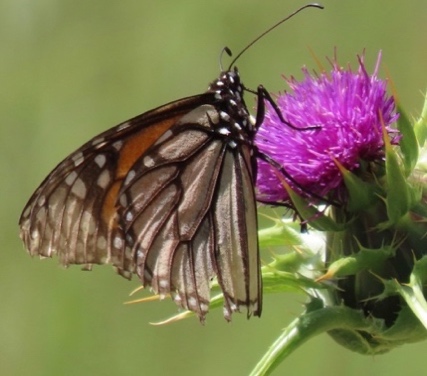 |  |

**References**

Caswell H. 2001. Matrix population models: Construction, analysis, and interpretation, 2nd edition. Sinaur, Sunderland, MA.

Crone EE, Schultz CB. 2022. Host plant limitation of butterflies in highly fragmented landscapes. Theoretical Ecology **15**:165–175.

Morris W., Doak D. 2002. Quantitative Conservation Biology : Theory and practice of population viability analysis. Oxford University Press, Oxford, UK.
